# The Association between Physical Health and Delusional-Like Experiences: A General Population Study

**DOI:** 10.1371/journal.pone.0018566

**Published:** 2011-04-25

**Authors:** Sukanta Saha, James Scott, Daniel Varghese, John McGrath

**Affiliations:** 1 Queensland Centre for Mental Health Research, The Park Centre for Mental Health, Wacol, Queensland, Australia; 2 Metro North Mental Health, Royal Brisbane and Women's Hospital, Hertson, Queensland, Australia; 3 Discipline of Psychiatry, School of Medicine, The University of Queensland, St Lucia, Queensland, Australia; 4 The University of Queensland Centre for Clinical Research, Herston, Queensland, Australia; 5 Princess Alexandra Hospital, Woolloongabba, Queensland, Australia; 6 Queensland Brain Institute, University of Queensland, St Lucia, Queensland, Australia; King's College London, United Kingdom

## Abstract

**Objective:**

Delusional-like experiences (DLE) are prevalent in the community. Recent community based studies have found that DLE are more common in those with depression and anxiety disorders, and in those with subclinical symptoms of depression and anxiety. Chronic physical disorders are associated with comorbid depression and anxiety; however, there is a lack of evidence about the association of DLE with common physical conditions. The aim of this study was to explore associations between the common physical disorders and DLE using a large population sample.

**Methods:**

Subjects were drawn from the Australian National Survey of Mental Health and Wellbeing 2007, a national household survey of 8841 residents aged between 16 and 85 years. The presence of DLE, selected common physical disorders and symptoms were assessed using a modified World Mental Health Composite International Diagnostic Interview (CIDI) schedule. We examined the relationship between DLE, and physical health-related variables using logistic regression, with adjustments for potential confounding factors.

**Results:**

Of the 8771, 776 (8.4%) subjects positively endorsed one or more DLE. Of the six physical disorders examined, only diabetes and arthritis were significantly associated with the endorsement of DLE. Of the seven broad physical symptoms explored, only hearing problems were consistently associated with DLE.

**Conclusion:**

Delusional-like experiences are common in the Australian community, and are associated with selected chronic physical disorders and with impaired hearing. The direction of causality between these variables warrants closer research scrutiny.

## Introduction

Several large community-based surveys have confirmed that many otherwise-well individuals report isolated delusional-like experiences (DLE) [1], [2]. A recent meta-analysis showed that the median prevalence of DLE was 3.5% of the population, with an inter-quartile range between 1.9 and 14.4% [3]. Apart from the well-recognized association with psychotic disorders [4], [5], there is also evidence that DLE are more prevalent in those with common mental disorders such as anxiety and depression, and in those exposed to traumatic life events [6]–[9]. Apart from clinical disorders, large community studies from the UK and the Netherlands have also reported increased risk of DLE in those who report symptoms of anxiety or depression [10], [11]. Given the association between DLE and these common mental disorders, it appears that DLE may be non-specific markers of psychological distress [12], [13].

It is well recognized that poor general physical health is associated with increased psychological distress and comorbid mental health disorders [14]–[16]. For example, physical disorders such as arthritis or diabetes are associated with distress and comorbid psychological problems [17]–[19]. In particular, disorders associated with sensory deprivation related to impairments in hearing or vision are associated with impaired mental health, including an increased risk of psychotic disorders [20]–[24]. It is feasible that those with chronic physical disorders may be at increased risk of DLE via their increased risk of general psychological distress and/or clinical disorders such as anxiety and depression. There is a lack of community-based research examining the association between common physical disorders and DLE. The aim of the present study was to examine the relationship between DLE, and physical illness/impairments using a large population study in Australia. Based on previous findings we hypothesised that individuals with chronic physical disorders or impairments were more likely to endorse DLE compared to those who were otherwise healthy.

## Methods

### 

#### Participants

Subjects were drawn from the Australian National Survey of Mental Health and Wellbeing 2007 (NSMHWB). Details of the survey methodology have been published elsewhere [25]. In brief, the NSMHWB was a national face-to-face household survey of 8841 community residents aged between 16 and 85 years. Sampling was based on random selection from a stratified, multistage area probability sample of private dwellings carried out by trained interviewers from the Australian Bureau of Statistics from August to December 2007. In total, 8,841 individuals participated in the survey.

#### Assessment of DSM-IV Diagnoses and Delusional-like Experiences

A modified version of the World Mental Health Survey Initiative of the Composite International Diagnostic Interview (WMH-CIDI 3.0) was used to generate DSM-IV lifetime diagnoses of depressive and anxiety disorders, alcohol and other drug abuse disorders [25], [26]. In keeping with our previous studies [2], [27] for the assessment of DLE, we used the items in Section G designed to screen for possible psychosis. Details of the DLE are given in Table S1. Briefly, DLE are composed of three ‘screen’ items followed by three ‘probe’ items. Those subjects who positively endorse any screen items are then asked a probe item. The items cover the following features of psychotic disorders: delusions of control, thought interference and passivity (Question 1 and 1a); delusions of reference or persecution (Question 2 and 2a); and grandiose delusions (Question 3 and 3a). Hallucination items are not included in the survey, and therefore not studied.

Also in keeping with our previous analyses [2], [9], [12], [27], [28], individuals who screened positive for schizophrenia (i.e. respondents who reported ‘Yes’ to the item “Had been told at any time by a psychiatrist that they had schizophrenia”) were excluded from the analyses (n = 68) leaving a total of 8 771 subjects for this study.

#### Assessment of physical disorders and impairment

The WMH-CIDI instrument includes two checklists related to the presence of physical disorders and impairments: (a) the National Health Priority Area (NHPA) physical illnesses [29], and (b) physical symptoms (henceforth ‘symptoms’). In the NHPA checklist, questions were asked with ‘yes’ or ‘no’ answers for six somatic illnesses: (a) asthma, (b) gout, rheumatism or arthritis (henceforth ‘arthritis’), (c) cancers, (d) diabetes or high blood sugar levels (henceforth ‘diabetes’), (e) any heart attack, angina or high blood pressure (henceforth ‘circulatory condition’), and (f) stroke or effects of stroke (henceforth ‘stroke’). Respondents were asked if the disorder (a) had occurred at any stage in the individual's life (lifetime), or (b) had been present in the 12 months prior to the interview. Respondents were also asked to confirm that they had these conditions for at least six months duration. With respect to physical impairments, respondents were asked about a range of symptoms including sight problems, hearing problems, restricted physical activity, difficulty in gripping, limited use of arms/fingers, and limited use of legs. These items were assessed for lifetime prevalence, but once again included the provision that the impairments persisted for at least six months.

#### Data analysis

For the main analyses, we examined the association between DLE (i.e. at least one of the G items was endorsed), and physical illnesses and symptoms using logistic regression where the first category (no physical illness/no symptoms/optimal health state) was used as the *reference* group. Based on previous research of factors associated with DLE [2], in Model 1, we adjusted for sex and age-at-testing. In response to previous studies that DLE are associated with anxiety and depressive disorders, alcohol and drug abuse/dependence, marital status, migration status, and family history of psychosis/schizophrenia [2], [26]–[28], we also examined a second model adjusting these potential confounding factors. As a planned sensitivity analysis, we restricted the screen items to two (Q1 & Q3), as it was possible that endorsement of screen item Q2 (‘ever felt people were too interested in you’) might be a direct result of having a severe chronic illness such as arthritis. In addition, as a *post-hoc* analysis, we adjusted the main results for education, socio-economic status (‘employment status’ as a proxy variable), and any traumatic events in life as there are well-known associations between physical health and socio-demographic status, and DLE and trauma [6].

Data were weighted to account for differential probability of selection and to adjust for over- or undersampling of population subgroups [25]. The initial weights were calibrated against known population estimates, and about 60 replicate weight variables were thus developed using the Jack-knife procedure of replication [30]. In the analyses, these weights (and replicate weights) were used to generate appropriate standard errors from which precise 95% confidence intervals were estimated. Analyses were performed using Proc *Surveylogistic* [31] which is designed to analyse complex survey samples using SAS (version 9.2;Cary, NC: SAS Institute). For distributions and measures of central tendency of physical health scores ‘Proc *surveyfreq*’ were used.

## Results

Of the 8,771 subjects included in the study, 776 (8.4%) positively endorsed one or more DLE screen items, and 295 (3.0%) endorsed one or more probe items. There was no significant sex difference in endorsing delusional-like experiences (Females 50.4%; OR: 1.21; 95% CI 0.97–1.50). The prevalence of the common physical conditions is shown in Table 1. The most prevalent physical condition was circulatory disorder (21%) followed by asthma and arthritis (about 20%), and cancers or diabetes (about 8%). As expected, the estimates for the last 12 months to the survey interview of these illnesses were lower than lifetime diagnoses (Table 1).

**Table 1:** Relationship between delusional-like experiences screen items and lifetime and 12 months physical illnesses (n = 8,771).

Physical disorders	Sample N (%, SE^1^)	Delusional-like experiences endorsement	
a. Lifetime		Model 1^2^ OR^4^(95%CI^5^)	Model 2^3^ OR^5^495%CI^5^)
**Arthritis@**	1987 (19.97, 0.60)	**1.58 (1.25, 1.99)** *	**1.35 (1.08, 1.72)** *
**Diabetes** $	693 (7.41, 0.39)	**1.94 (1.35, 2.78)** *	**1.93 (1.33, 2.81)** *
**Asthma**	1765 (19.42, 0.55)	1.17 (0.94, 1.44)	1.09 (0.87, 1.37)
**Cancers**	881 (8.30, 0.36)	1.13 (0.83, 1.71)	1.08 (0.75, 1.57)
**Stroke** &	231 (2.00, 0.13)	**2.13 (1.18, 3.84)** *	1.46 (0.75, 2.84)
**Circulatory condition^#^**	2046 (21.18, 0.68)	1.16 (0.84, 1.61)	1.07 (0.77, 1.49)

b. 12 months			
**Arthritis@**	1288 (12.74, 0.41)	**1.70 (1.30, 2.23)** *	**1.46 (1.12, 1.89)** *
**Diabetes** $	480 (4.93, 0.30)	**2.23 (1.49, 3.31)** *	**2.14 (1.40, 3.25)** *
**Asthma**	783 (8.62, 0.42)	1.33 (0.97, 1.83)	1.25 (0.91, 1.73)
**Cancers**	283 (2.81, 0.25)	1.38 (0.71, 2.66)	1.23 (0.59, 2.53)
**Stroke** &	67 (0.55, 0.08)	**3.37 (1.51, 7.52)** *	2.35 (0.85, 6.48)
**Circulatory condition** %	1427 (14.82, 0.60)	0.88 (0.66, 1.17)	0.85 (0.63, 1.15)

1 SE = Standard error of the frequency.

2 Model 1 adjusted for age and sex;

3 Model 2 = adjusted for age, sex, marital status, migrant status, any alcohol abuse/dependence, any illicit drug abuse/dependence, any anxiety disorders, any depressive disorders, and family history of schizophrenia/psychosis.

4 OR = Odds Ratio,

5 CI = Confidence Interval

*significance: *p*<0.01 (shown in bold).

@ Arthritis = gout, rheumatism or arthritis,

$ Diabetes = diabetes or high blood sugar,

& Stroke = stroke or effects of stroke,

% Circulatory condition = heart attack, angina or high blood pressure.

The relationship between the selected physical illnesses and DLE as assessed by Screen items are shown in Table 1, while those assessed by Probe items are shown in Table 2. Those with lifetime diagnoses of diabetes and arthritis were significantly more likely to endorse both screen and probe items compared with those who had not reported these illnesses. This relationship was present in those who reported the presence of the physical conditions over their lifetime and for the past year. Although the results were generally comparable for screen and probe items, the association between asthma and DLE reached significance level for probe items (OR = 1.79, 95% CI: 1.09, 2.95). These associations remained statistically significant after adjusting for potential confounding factors (including the presence of anxiety and depressive disorders). The point estimates fell slightly when adjusted for the presence of common mental disorders such as depression and anxiety. None of the other physical disorders was significantly associated with DLE.

**Table 2:** Relationship between delusional-like experience probe items and lifetime and 12 months physical illnesses (n = 8,771).

Physical disorders	Delusional-like experiences endorsement	
a. Lifetime	Model 1^1^ OR^3^(95%CI^4^)	Model 2^2^ OR^3^495%CI^4^)
**Arthritis@**	**1.79 (1.19, 2.67)** *	**1.53 (1.03, 2.28)** *
**Diabetes** $	**1.79 (1.22, 2.62)** *	**1.77 (1.15, 2.73)** *
**Asthma**	**1.43 (1.00, 2.04)** *	1.33 (0.92, 1.92)
**Cancers**	**1.53 (1.03, 2.27)** *	1.48 (0.99, 2.21)
**Stroke** &	**2.59 (1.14, 5.91)** *	1.81 (0.70, 4.67)
**Circulatory condition^#^**	1.32 (0.89, 1.96)	1.23 (0.80, 1.88)

b. 12 months		
**Arthritis** @	**1.93 (1.22, 3.06)** *	**1.65 (1.03, 2.64)** *
**Diabetes** $	**2.02 (1.32, 3.09)** *	**1.92 (1.20, 3.08)** *
**Asthma**	**1.79 (1.09, 2.95)** *	**1.67 (1.01, 2.77)** *
**Cancers**	1.39 (0.73, 2.65)	1.40 (0.76, 2.61)
**Stroke** &	**4.63 (1.41, 15.24)** *	3.42 (0.82, 14.3)
**Circulatory condition** %	1.11 (0.67, 1.83)	1.12 (0.67, 1.89)

1 Model 1 adjusted for age and sex;

2 Model 2 = adjusted for age, sex, marital status, migrant status, any alcohol abuse/dependence, any illicit drug abuse/dependence, any anxiety disorders, any depressive disorders, and family history of schizophrenia/psychosis.

3 OR = Odds Ratio,

4 CI = Confidence Interval

*significance: *p*<0.01 (shown in bold).

@ Arthritis = gout, rheumatism or arthritis,

$ Diabetes = diabetes or high blood sugar,

& Stroke = stroke or effects of stroke,

% Circulatory condition = heart attack, angina or high blood pressure.

Concerning physical symptoms, the most prevalent symptom was hearing problem (about 9%), while about 6% reported sight problems and restricted physical activity (see Table 3). Table 3 and 4 shows the relationship between the physical symptoms and DLE. Among these impairments, only subjects with sight and hearing problems were more likely to endorse screen items compared to those with no impairments (sight OR = 2.01, 95% CI: 1.37, 2.96; hearing OR = 1.52, 95% CI: 1.12, 2.06). Once again, while the point estimates fell in the model adjusting for the presence of comorbid depression or anxiety disorders, these associations remained statistically significant. For probe items, only hearing problems remained statistically significant (Table 4).

**Table 3:** Relationship between delusional-like experience screen items and physical symptoms (n = 8,771).

Symptoms	Sample N (%, SE^1^)	Delusional-like experiences endorsement	
		Model 1^2^ OR^4^(95%CI^5^)	Model 2^3^ OR^4^(95%CI^5^)
**Sight problem**	593 (5.97, 0.32)	**2.01 (1.37, 2.96)** *	**1.64 (1.11, 2.41)** *
**Hearing problem**	882 (8.98, 0.48)	**1.52 (1.12, 2.06)** *	**1.40 (1.00, 1.97)** *
**Difficulty in learning**	102 (1.02, 0.11)	**1.78 (1.00, 3.16)** *	0.99 (0.53, 1.88)
**Restricted physical activity**	514 (5.91, 0.35)	1.30 (0.89, 1.910	1.08 (0.70, 1.66)
**Difficulty in gripping**	107 (1.09, 0.12)	1.93 (0.86, 4.32)	1.60 (0.72, 3.57)
**Limited use of arms/fingers**	210 (2.46, 0.23)	**2.08 (1.17, 3.70)** *	1.66 (0.98, 2.84)
**Limited use of Legs**	153 (1.27, 0.11)	1.26 (0.73, 2.17)	0.97 (0.54, 1.74)

1 SE = Standard error of the frequency.

2 Model 1 adjusted for age and sex;

3 Model 2 = adjusted for age, sex, marital status, migrant status, any alcohol abuse/dependence, any illicit drug abuse/dependence, any anxiety disorders, any depressive disorders, and family history of schizophrenia/psychosis.

4 OR = Odds Ratio,

5 CI = Confidence Interval.

*significance: *p*<0.01 (shown in bold).

**Table 4:** Relationship between delusional-like experience probe items and physical symptoms (n = 8,771).

Symptoms	Delusional-like experiences endorsement	
	Model 1^1^ OR^3^(95%CI^4^)	Model 2^2^ OR^3^(95%CI^4^)
**Sight problem**	1.48 (0.76, 2.86)	1.09 (0.54, 2.18)
**Hearing problem**	**1.90 (1.17, 3.09)** *	**1.78 (1.08, 2.92)** *
**Difficulty in learning**	**3.38 (1.49, 7.63)** *	1.72 (0.76, 3.93)
**Restricted physical activity**	**2.04 (1.02, 4.09)** *	1.49 (0.73, 3.05)
**Difficulty in gripping**	1.63 (0.59, 4.53)	1.29 (0.48, 3.44)
**Limited use of arms/fingers**	**3.47 (1.42, 8.51)** *	2.61 (0.97, 6.99)
**Limited use of Legs**	**2.42 (1.28, 4.55)** *	1.77 (0.89, 3.55)

1 Model 1 adjusted for age and sex;

2 Model 2 = adjusted for age, sex, marital status, migrant status, any alcohol abuse/dependence, any illicit drug abuse/dependence, any anxiety disorders, any depressive disorders, and family history of schizophrenia/psychosis.

3 OR = Odds Ratio,

4 CI = Confidence Interval.

*significance: *p*<0.01 (shown in bold).

In a planned sensitivity analysis where we restricted the DLE screen items to Q1 and Q3 only, the results remained essentially unchanged (data not shown). The results also remained unchanged in the *post-hoc* analysis where further adjustment was considered with education, socio-economic status and trauma variables (data not shown).

## Discussion

Individuals who report physical disorders including arthritis, gout or rheumatism, and diabetes or high blood sugar were more likely to endorse DLEs compared to those who were otherwise healthy, and this relationship persisted when we adjusted for the presence of common mental conditions such as anxiety disorder or depression. Individuals with arthritis have high scores for pain, physical immobility, impaired sleep and depression [17], [32]. It is feasible that individuals with arthritis may have heightened distress for a long period of time, which may subsequently lead to the emergence of DLEs [33], [34]. Similarly, it is also possible that individuals with diabetes may have underlying psychological distress because of its' chronic nature. Studies show that patients of both Type 1 [35]–[37] and Type II [19] diabetes suffer from psychological distress.

With respect to physical impairments, our study found that individuals with sensory impairments but not other physical impairments were more likely to endorse DLE screen items compared to those who did not have these problems. This is congruent with the report that the hearing impairment is associated with an increased risk of psychotic symptoms in both old [20]–[22] and young patients, and also in clinical and non-clinical subjects [23], [24].

In our study certain physical disorders and selected symptoms are independently associated with DLE regardless of a range of confounding factors including CIDI diagnoses of depressive and anxiety disorders, alcohol and drug abuse and/or dependence, trauma, socio-demographic variables such as migrant status, marital status, employment, or educational status, and family history of psychosis/schizophrenia. This analysis suggests that the association between physical illness and symptoms is not mediated by the presence of these risk factors.

The study has several limitations that merit caution in interpreting the results. The study is cross-sectional; therefore, we cannot comment on the direction of causality. Our results indicate that those who have physical illnesses such as arthritis, diabetes, and those who have physical symptoms such as sight and hearing impairment are more likely to endorse DLE. While we cannot exclude the possibility, it seems less likely that pre-existing DLE might subsequently lead to the onset of co-morbid physical conditions such as arthritis and sensory motor impairment (i.e. reverse causality). While individuals with schizophrenia are at increased risk of a range of co-morbid physical conditions related to life style and medication [38], [39] those with a past history of schizophrenia were excluded from the analyses. We used only three screen items to measure delusional-like experiences and there were no items for hallucinations. However, previous studies have shown a strong association between DLE and hallucinations in general population samples [1], [40]. For the main explanatory variables, we had to rely on an individual's response on physical and psychiatric illnesses and symptoms for lifetime and 12 months respectively, and we were not able to validate these self-reported diagnoses. As recent studies indicate that lifetime diagnoses for psychiatric illnesses underreport true prevalence estimates [41], [42], the lifetime estimates for various conditions may be inaccurate. However when we examined the more reliable past-year estimates, the point estimates for most disorders increased. Because the data were obtained from a household survey some population groups such as homeless, people living in nursing home, hostels etc were not surveyed.

Our study provides evidence that arthritis, diabetes and hearing impairment are associated with delusional-like experiences in the general population. Interest is steadily growing in understanding the clinical utility of DLEs for identification of individuals at greater risk of mental disorders such as anxiety, depression and psychotic disorders. Our study suggests that pathways related to physical health may also contribute to the presence of DLE, perhaps via associated general psychological distress [12]. Longitudinal studies may be able to provide insights into the causal pathways that underpin the associations found in this population-based study.

## Supporting Information

Table S1 CIDI Screen and Probes items for Psychosis (Delusional-like experiences, DLE).
(DOCX)
