# Supplementary material for: The Association between Physical Health and Delusional-Like Experiences: A General Population Study
Source: PLoS One. 2011 Apr 25;6(4):e18566. doi: 10.1371/journal.pone.0018566 (PMC3081831; doi:10.1371/journal.pone.0018566)
Supplement: Table S1 — CIDI Screen and Probes items for Psychosis (Delusional-like experiences, DLE). (DOCX) [file pone.0018566.s001.docx]

**Table S1. CIDI Screen and Probes items for Psychosis (Delusional-like experiences, DLE)**

| **Item G1 (PS1*):** |
| --- |
| Have you ever felt that your thoughts were being directly interfered with or controlled by another person? |
| **If yes, PS1A^#^:** |
| Did it come about in a way that many people would find hard to believe, for instance, through telepathy? |
| **Item G2 (PS2*):** |
| Have you ever had a feeling that people were too interested in you? |
| **If yes, PS2A^#^:** |
| Have you had a feeling that things were arranged so as to have a special meaning for you, or even that harm might come to you? |
| **Item G1 (PS3*):** |
| Do you ever have any special powers that most people lack? |
| **If yes, PS3A^#^:** |
| Do you belong to a group of people who also have these powers? |
|  |

Item PS4^@^:

Has a doctor ever told you that you may have schizophrenia?

*Screen items (lifetime) with answer (Yes/No): ‘Any screen’ items required ‘Yes’ answers to all three questions.

#Probe items (lifetime) with answer (Yes/No):. ‘Any probe’ items required ‘Yes’ answers to PS1A and PS2A, and ‘No’ answer to PS3A.

^@^Sample excluded from the analyses (n=68)
